# Supplementary material for: Predictive and robust gene selection for spatial transcriptomics
Source: Nat Commun. 2023 Apr 12;14:2091. doi: 10.1038/s41467-023-37392-1 (PMC10097645; doi:10.1038/s41467-023-37392-1)
Supplement: Supplementary file 1 — Supplementary Information [file 41467_2023_37392_MOESM1_ESM.pdf]

Supplementary Note 1

This note provides additional results for evaluating PERSIST. First, we present results for the expression profile reconstruction metrics using the supervised methods, scGeneFit, SMaSH and MutInfo, which leverage cell type labels when selecting genes. Supp. Fig. 3 shows their explained variance and expressed gene prediction accuracy for the SSv4 and 10X datasets. MutInfo is competitive for small gene panels, but none of the three methods matches PERSIST's performance.

Next, we examine whether introducing binarization into the baseline methods enables better performance on our evaluation metrics, which simulate the use of gene panels in a FISH study. Supp. Fig. 4 shows that binarization makes scGeneFit, SMaSH and GeneBasis more competitive with PERSIST, and that they in some cases even match PERSIST's performance. In contrast, binarization makes Seurat and Cell Ranger perform worse, which is unsurprising because variance and dispersion values are significantly less informative for binary data. Overall, even when all the gene selection methods used binarized data, PERSIST is either the best or tied for best on each metric, suggesting better transferability to FISH studies.

To give a more granular view of PERSIST's expressed gene prediction accuracy, we provide additional plots with gene-level accuracy metrics. As in the main text, we calculate accuracy according to how often the prediction agrees with each gene's detection in the data, but we now report results for each individual gene in the SSv4 dataset. Supp. Fig. 5 shows results for the PERSIST panels containing 32 and 128 genes, with the per-gene accuracy plotted against the portion of cells in which the gene is expressed, and against the average expression level (after log-CPM normalization). The results show that the most difficult genes to predict are those which are neither ubiquitously expressed or not expressed, and which have moderate mean expression. There is a visible improvement when we use a panel of 128 rather than 32 genes, but even then, the prediction problem remains difficult due to stochasticity in gene expression and detection.

In the main text, the expressed gene prediction results focus on genes that are expressed in 20-80% of cells, ignoring the remaining genes whose expression is easiest to predict. The results with different ranges of 10-90% and 0-100% are shown in Supp. Fig. 6. The trend between methods does not depend on the cutoff, but including the easier genes increases the overall accuracy while shrinking the gap between methods.

Regarding cell type classification, the main text reports accuracy results averaged across all cell types, so we now provide additional results that give a more granular view. In Supp. Fig. 7, we present two results for the PERSIST panel containing 32 genes from the SSv4 dataset: a confusion matrix showing how cells of each type are classified, and the recall (true positive rate) shown for each cell type. The results show that while some cell types are mostly correctly classified, some have relatively low recall, reflecting the imperfect overall accuracy (63%). In Supp. Fig. 8, we show the same results but for the PERSIST-Classification panel containing 32 genes. The results are visibly improved, reflecting the higher overall accuracy (74%), but certain cell types remain difficult to classify. This provides further motivation for designing FISH studies that focus on coarse-grained cell types, as we described in the main text.

Next, as an intuitive visualization of how PERSIST can distinguish cell types using only binarized expression levels, we plot the frequency of each gene being expressed within each cell type. Supp. Fig. 9 displays this expression matrix for the full set of transcriptomic cell types in the SSv4 dataset, as well as for 50 and 25 subclasses, using the PERSIST and PERSIST-Classification panels containing 16 genes. The results show that each cell type has a unique expression pattern, enabling us to distinguish cell types using a surprisingly small number of genes. Although all configurations of gene expression are technically possible, we find that many genes are expressed nearly all of the time or none of the time within each cell type. For example, we find that with all 113 transcriptomic cell types, 67% of the entries are expressed

either less than 20% or more than 80% of the time (Supp. Fig. 9A); this figure is even higher at 74% for PERSIST-Classification (Supp. Fig. 9D).

For the MERFISH gene imputation experiments, we provide several additional results in Supp. Fig. 10. First, Supp. Figs. 10A-B show the imputation accuracy for the supervised methods, scGeneFit, SMaSH and MutInfo. We find that when using the SSv4 V1 scRNA-seq data, PERSIST and MutInfo reach comparable accuracy for small gene panels, but that PERSIST achieves higher accuracy with larger panels; when using the SSv4 ALM scRNA-seq data, the various methods offer comparable accuracy for panels of all sizes. Next, we examine the importance of using a carefully chosen threshold when binarizing the MERFISH gene expression counts. If we naively use a threshold value of zero, the imputation accuracy is significantly lower than if we choose a threshold to match the quantile that zero represents in the scRNA-seq data (Supp. Fig. 10C). Finally, we use the fully observed MERFISH dataset to quantify the accuracy lost due to training on out-of-domain scRNA-seq data. We find that training with the in-domain MERFISH data yields an accuracy improvement of 4-6% (Supp. Fig. 10D), suggesting that our binarization step does not completely remove the effects of the domain shift relative to the SSv4 V1 scRNA-seq data.

Regarding the diversity in gene panels between different methods, in addition to the results for panels of 32 genes (Fig. 6), we report the gene panel overlap for panels of size 16 and 128 in Supp. Fig. 11. For panels of these sizes, we still find that PERSIST selects genes that are largely different than those identified by other methods. We also find several sets of similar methods that appeared in the main text: scGeneFit and GeneBasis, Seurat and Cell Ranger, and MutInfo, SMaSH and PERSIST-Classification.

Similarly, we report gene panel overlap for the experiments with Patch-seq data (Supp. Fig. 12). The results show that PERSIST-Ephys selects up to roughly 30% of the same genes as other methods, with Cell Ranger being the most similar for all panel sizes. PERSIST and Cell Ranger are the next most effective methods in this experiment after PERSIST-Ephys, but these three methods select largely distinct gene panels.

Finally, we examine to what extent the variability in PERSIST's selections across trials impacts the performance in our evaluation metrics (Supp. Fig. 13). Using five independent trials with the SSv4 dataset, we examine the mean performance across trials, the gap between the minimum and maximum performance, and the performance from the single trial chosen according to its validation loss (calculated using the PERSIST reconstruction model's hurdle loss). We find that the variability in performance is relatively small, particularly for the explained variance results (Supp. Fig. 13A). Choosing a single trial based on its validation loss tends to result in performance above the mean for smaller panels ($\leq$16 genes), but with larger panels it can be slightly less effective than choosing a single trial at random. These results suggest that the significant redundancy in genome-wide expression profiles enables the selection of diverse panels with nearly equivalent information content.

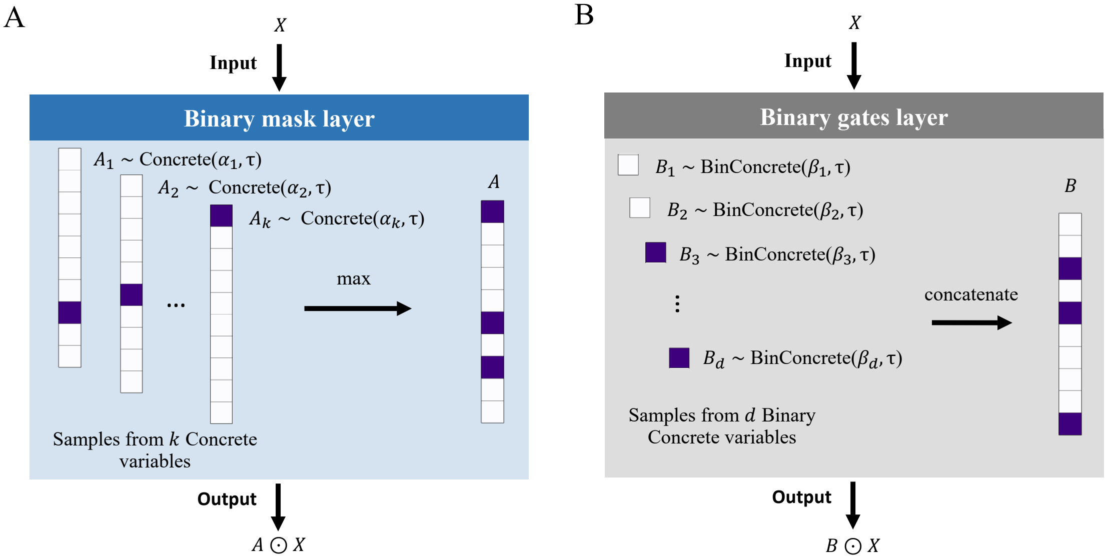

Supplementary Figure 1: **Feature selection layers.** **A**, The binary mask layer multiplies its input with a learned, k-hot binary mask whose entries are the element-wise maximum of samples from k Concrete random variables. **B**, The binary gates layer multiplies the input with a learned binary mask whose entries are samples from d separate BinConcrete random variables.

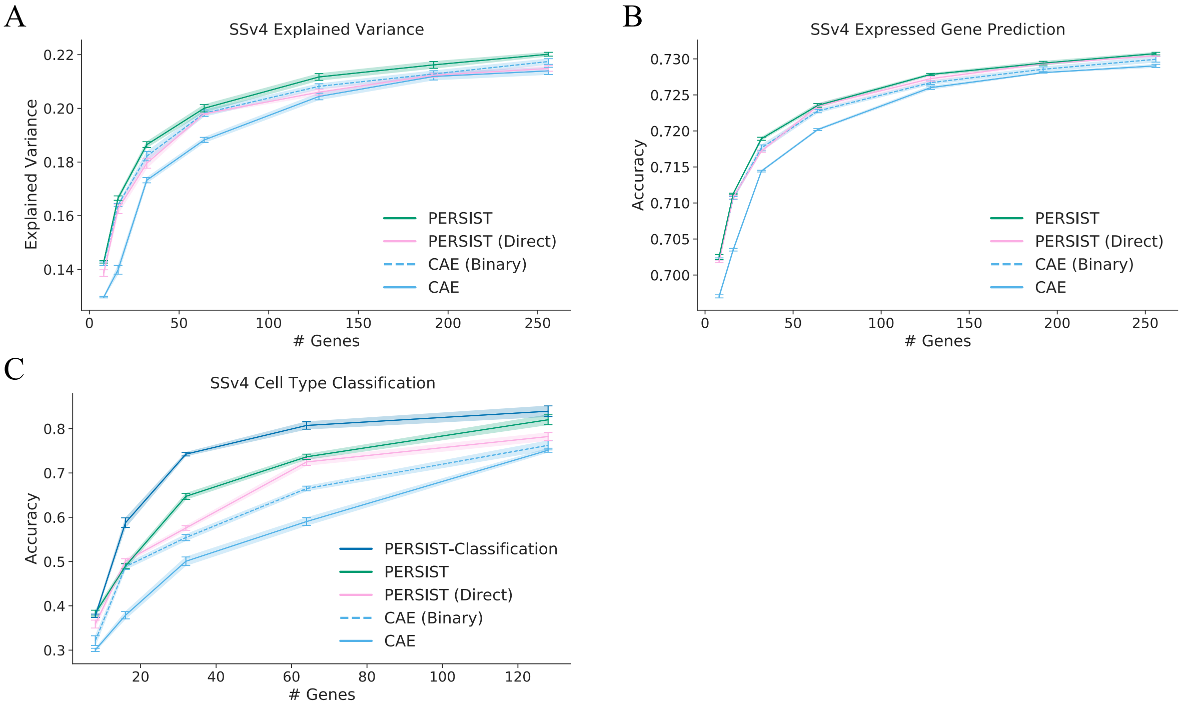

Supplementary Figure 2: **PERSIST ablations.** PERSIST performs better than the CAE on expression profile reconstruction tasks and cell type classification. The performance gap narrows when the CAE uses binarized expression counts, but the difference in cell type classification accuracy remains large. PERSIST also performs slightly better when initially narrowing the set of candidate genes, rather than proceeding directly to training with the binary mask layer (PERSIST Direct). **A**, Explained variance for the SSv4 dataset. **B**, Expressed gene prediction accuracy for the SSv4 dataset. **C**, Cell type classification accuracy for the SSv4 dataset. Results were calculated using a set of n=2,216 held-out cells, and all error bars represent 95% confidence intervals determined by training with five bootstrapped datasets.

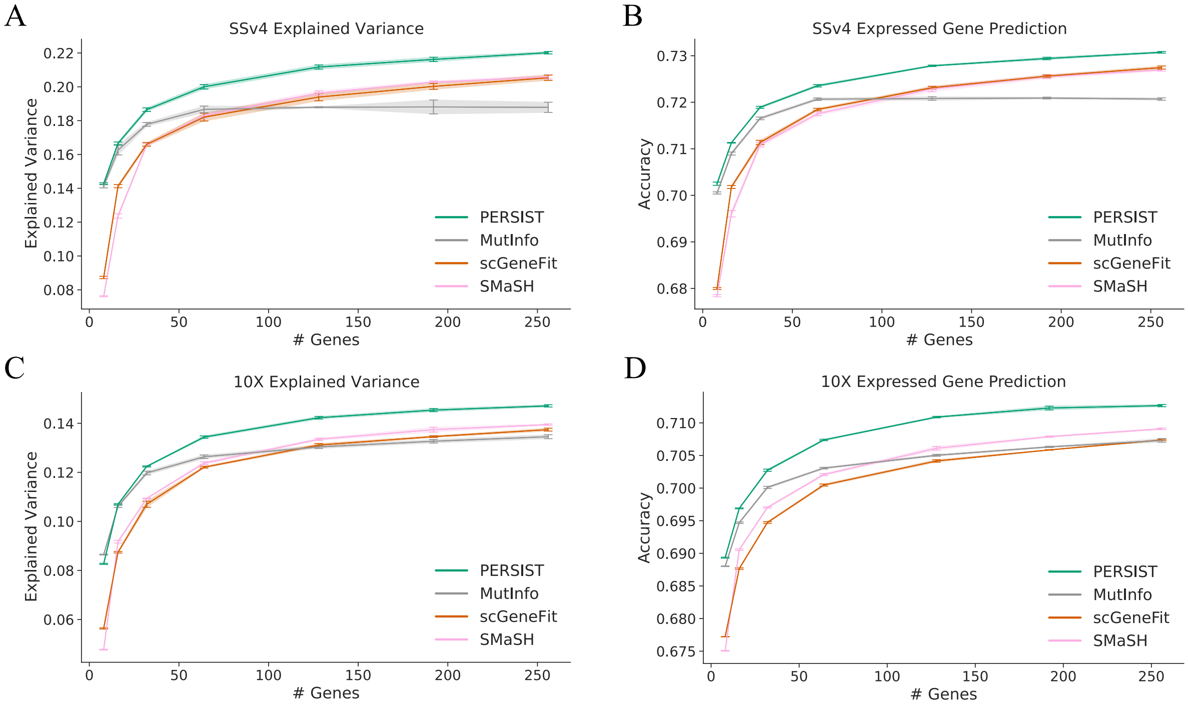

Supplementary Figure 3: **Expression profile reconstruction with supervised selection methods.** We compare PERSIST with the two supervised methods that leverage cell type labels, scGeneFit and MutInfo, on the tasks of predicting log-normalized expression counts (**A**, **C**) and whether each gene is expressed (**B**, **D**). **A**, Explained variance for gene panels with the SSv4 dataset. **B**, Expressed gene prediction accuracy for the SSv4 dataset. **C**, Explained variance for gene panels with the 10X dataset. **D**, Expressed gene prediction accuracy for the 10X dataset. The results were calculated using a set of held-out cells from each dataset, with n=2.216 for the SSv4 cells (**A**-**B**) and n=7,262 for the 10X cells (**C**-**D**). All error bars represent 95% confidence intervals determined by training with five bootstrapped datasets.

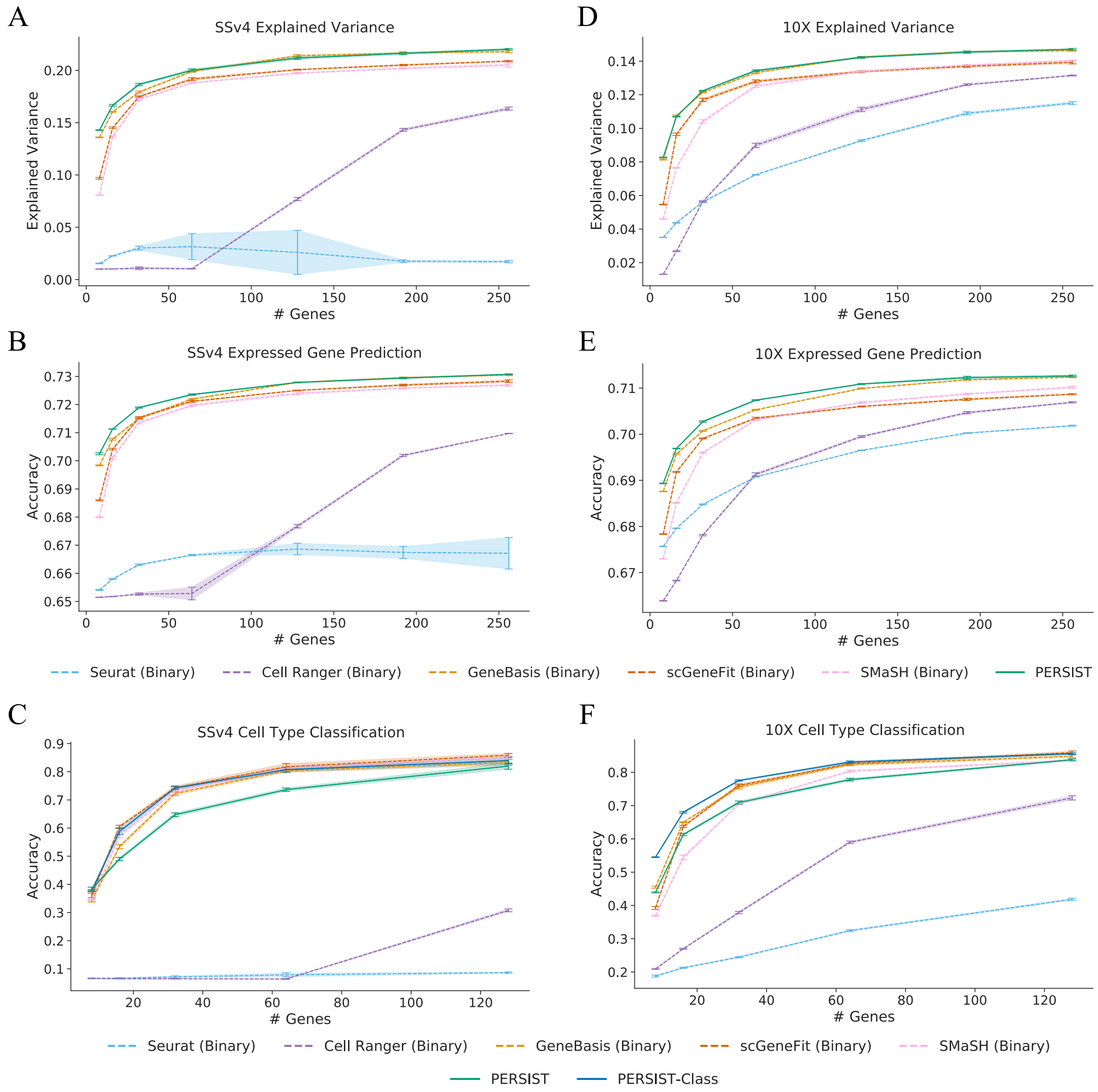

Supplementary Figure 4: **Binarizing gene expression for existing methods.** Binarizing gene expression levels before selecting the gene panel enable certain baselines to perform better in our evaluation. GeneBasis, SMaSH and scGeneFit become more competitive with PERSIST and PERSIST-Classification, while Seurat and Cell Ranger perform worse. **A**, Explained variance for gene panels with the SSv4 dataset. **B**, Expressed gene prediction accuracy for the SSv4 dataset. **C**, Cell type classification accuracy for the SSv4 dataset. **D**, Explained variance for the 10X dataset. **E**, Expressed gene prediction for the 10X dataset. **F**, Cell type classification accuracy for the 10X dataset. The results were calculated using a set of held-out cells from each dataset, with n=2.216 for the SSv4 cells (**A**-**C**) and n=7,262 for the 10X cells (**D**-**F**). All error bars represent 95% confidence intervals determined by training with five bootstrapped datasets.

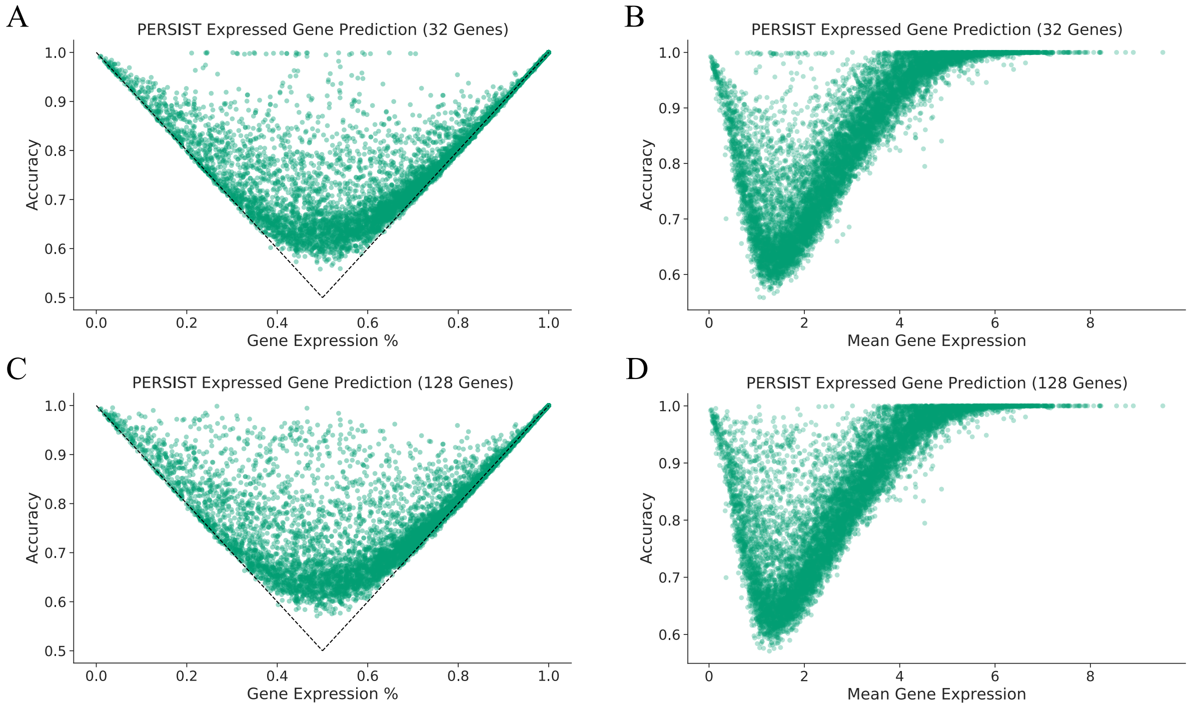

Supplementary Figure 5: **PERSIST expressed gene prediction.** The accuracy when predicting each gene's expression is plotted against the gene's characteristics. Genes that are either frequently expressed or infrequently expressed are easiest to predict (**A**, **C**), and those with moderate mean expression are most difficult to predict (**B**, **D**). **A**, Prediction accuracy versus the percentage of cells in which the gene is expressed, for the PERSIST panel containing 32 genes. Dotted lines indicate the accuracy achieved by trivially predicting that each gene is either always expressed or never expressed. **B**, Prediction accuracy versus mean expression level, for the 32-gene PERSIST panel. **C**, Prediction accuracy versus the percentage of cells in which the gene is expressed for the 128-gene PERSIST panel. **D**, Prediction accuracy versus mean expression level for the 128-gene PERSIST panel.

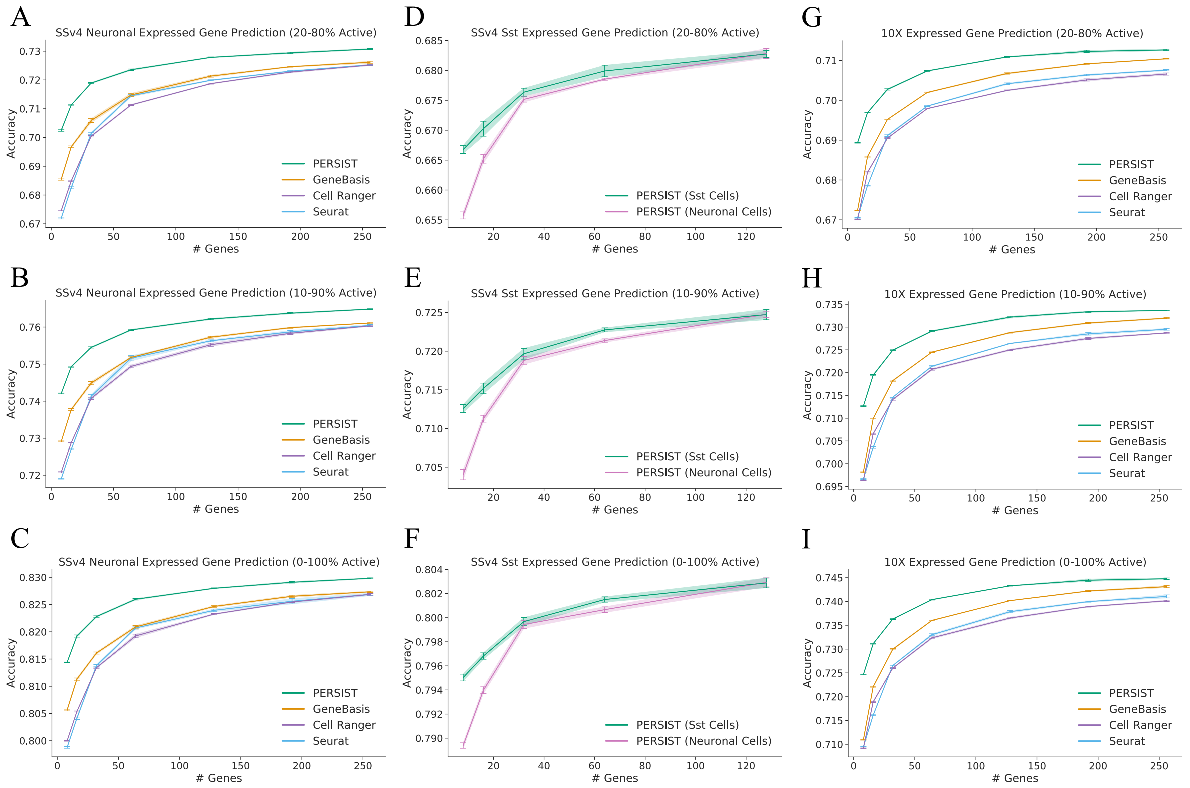

Supplementary Figure 6: **Expressed gene prediction with different expression cutoffs.** Genes that are expressed in either all cells or no cells are easiest to predict, and focusing on these genes can inflate the expressed gene prediction accuracy. We examine how the accuracy changes as we apply different cutoffs to focus on more difficult-to-predict genes. **A**, SSv4 accuracy for genes expressed in 20-80% of cells. **B**, SSv4 accuracy for genes expressed in 10-90% of cells. **C**, SSv4 accuracy for genes expressed in 0-100% of cells (all genes). **D**, SSv4 Sst accuracy for genes expressed in 20-80% of cells. **E**, SSv4 Sst accuracy for genes expressed in 10-90% of cells. **F**, SSv4 Sst accuracy for genes expressed in 0-100% of cells. **G**, 10X accuracy for genes expressed in 20-80% of cells. **H**, 10X accuracy for genes expressed in 10-90% of cells. **I**, 10X accuracy for genes expressed in 0-100% of cells (all genes). The results were calculated using a set of held-out cells from each dataset, with n=2,216 for the SSv4 neuronal cells (**A**-**C**), n=270 for the SSv4 Sst cells (**D**-**F**), and n=7,262 for the 10X cells (**G**-**I**). All error bars represent 95% confidence intervals determined by training with five bootstrapped datasets.

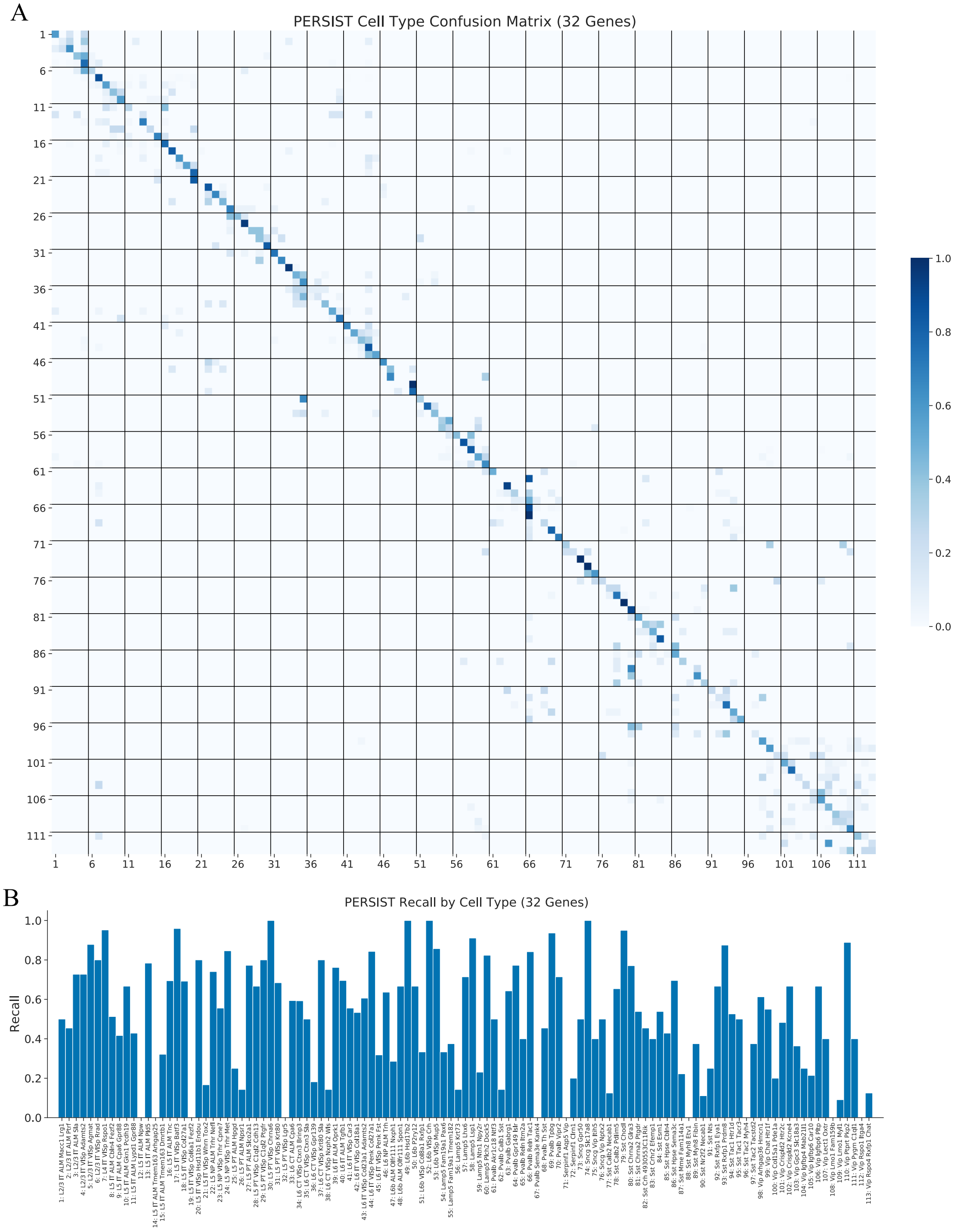

Supplementary Figure 7: **PERSIST cell type classification metrics.** Additional metrics quantify the classification accuracy on a per-type level for the 32-gene PERSIST panel. **A**, Confusion matrix where each row corresponds to a cell type and values indicate the percentage of cells classified into each type. Names for each numbered cell type are provided in the bar chart below. **B**, Recall (true positive rate) for each cell type.

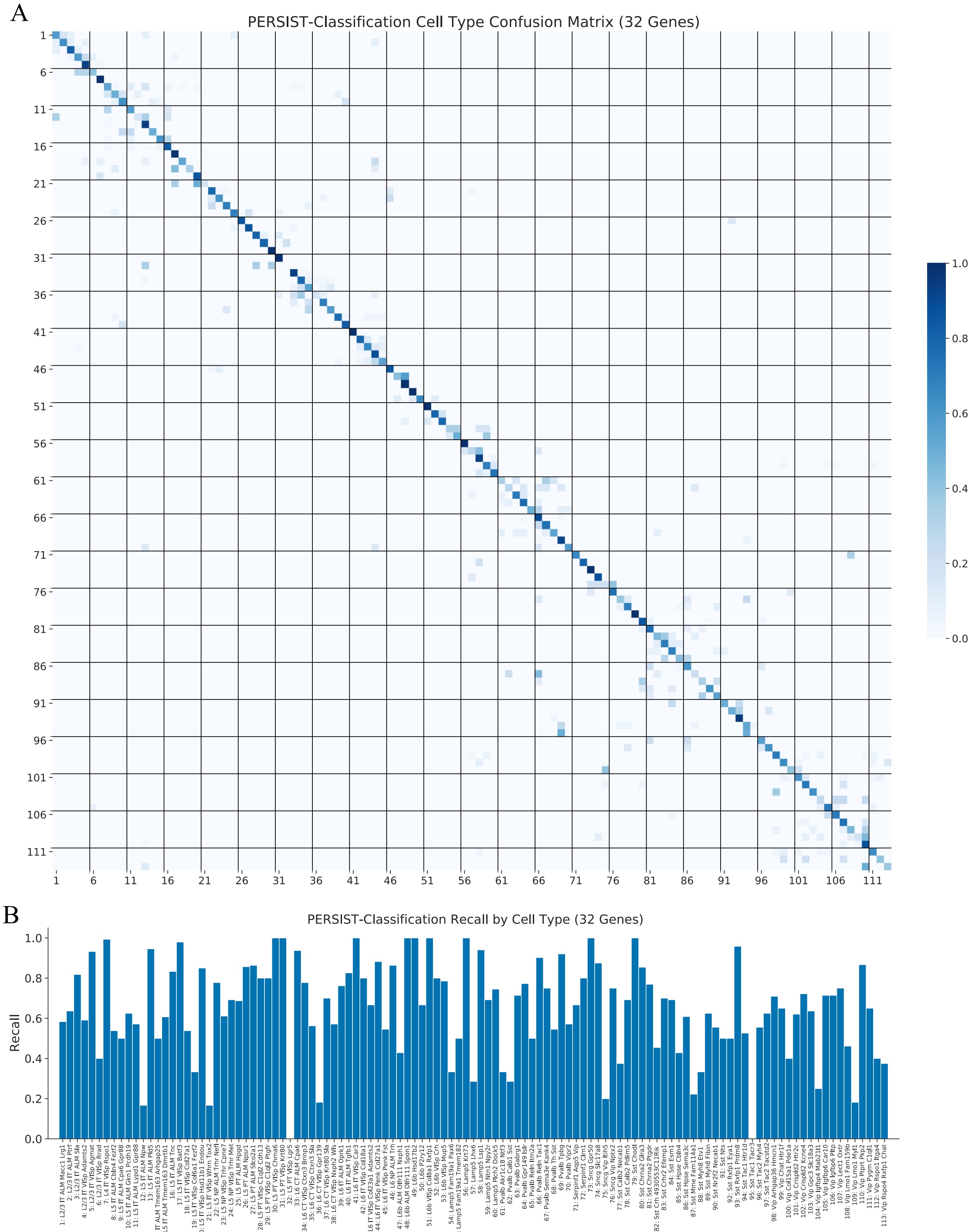

Supplementary Figure 8: **PERSIST-Classification cell type classification metrics.** Additional metrics quantify the classification accuracy on a per-type level for the 32-gene PERSIST-Classification panel. **A**, Confusion matrix where each row corresponds to a cell type and values indicate the percentage of cells classified into each type. Names for each numbered cell type are provided in the bar chart below. **B**, Recall (true positive rate) for each cell type.

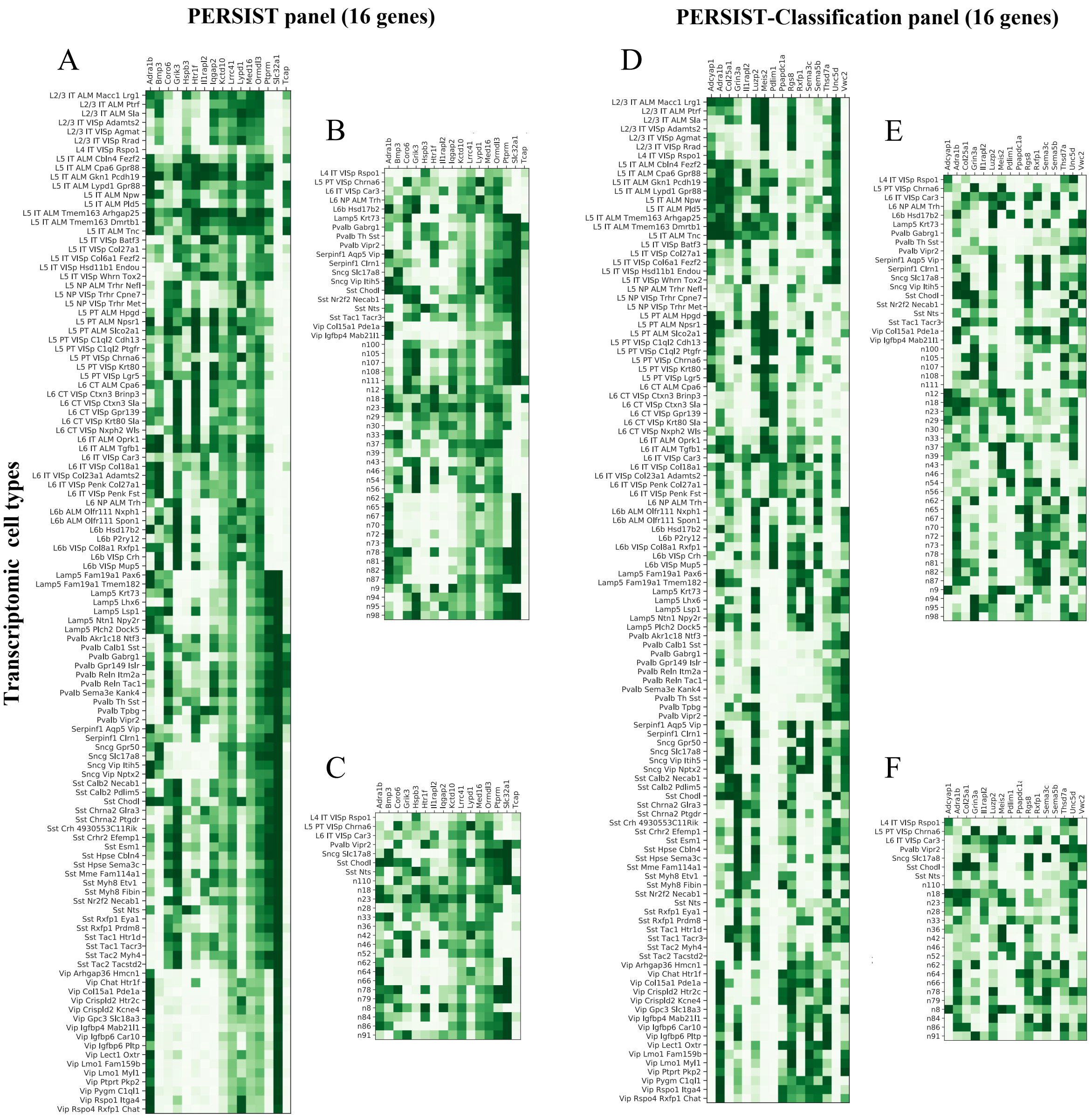

Supplementary Figure 9: **PERSIST gene expression frequency by cell type.** Using the SSv4 dataset, we plot the portion of the time that each gene in a panel is expressed within each cell type (white indicates never expressed, dark green indicates always expressed). Examining these matrices for the PERSIST and PERSIST-Classification panels containing 16 genes, using both the full set of transcriptomic cell types as well as small numbers of subclasses, we can identify distinct expression patterns within each cell type. **A**, Expression matrix for PERSIST gene panel with all cell types. **B**, Expression matrix for PERSIST gene panel with 50 subclasses. **C**, Expression matrix for PERSIST gene panel with 25 subclasses. **D**, Expression matrix for PERSIST-Classification gene panel with all cell types. **E**, Expression matrix for PERSIST-Classification gene panel with 50 subclasses. **F**, Expression matrix for PERSIST-Classification gene panel with 25 subclasses.

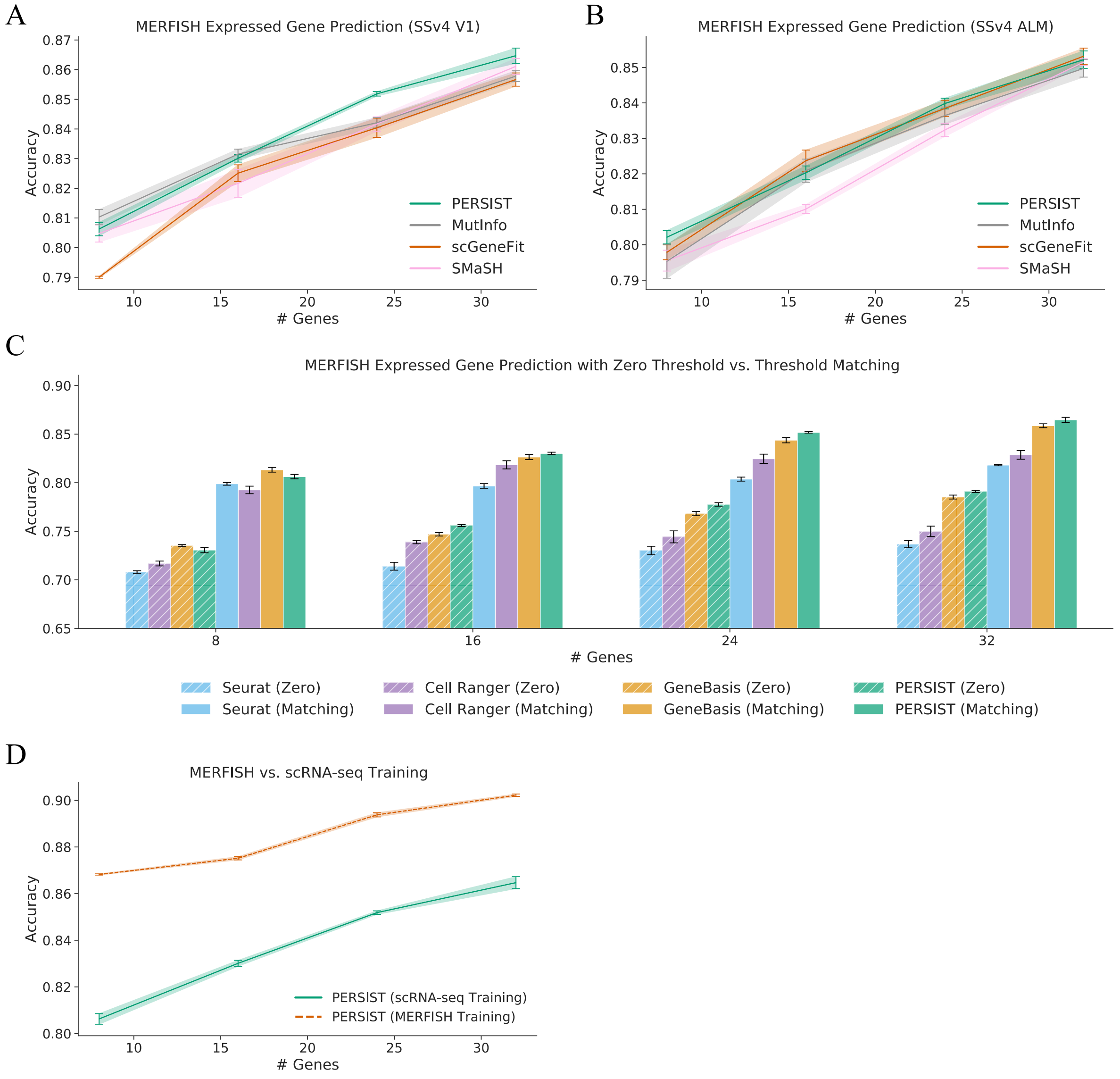

Supplementary Figure 10: **Additional MERFISH imputation results.** Here, we report results for the supervised gene selection methods, investigate the importance of the thresholding procedure for MERFISH expression counts, and examine the accuracy lost due to domain shift between the scRNA-seq and MERFISH datasets. **A**, Imputation accuracy for panels selected by each method when using the SSv4 V1 scRNA-seq data. PERSIST narrowly outperforms the supervised baselines, except for MutInfo with small panel sizes. **B**, Imputation accuracy for panels selected by each method when using the SSv4 ALM scRNA-seq data. The various methods achieve similar performance. **C**, Imputation accuracy when using a zero threshold for MERFISH expression counts, versus when using a threshold that matches the scRNA-seq quantile. Matching the threshold significantly improves the accuracy for all methods across all panel sizes. **D**, Comparing imputation accuracy when selecting genes using V1 cells but training on V1 scRNA-seq versus MOp MERFISH data. Training on in-domain MERFISH data, which is free of domain shift but inaccessible in practice, reveals an accuracy loss of 4-6% due to training on scRNA-seq. The results when training with SSv4 cells were calculated using all n=280,327 MERFISH cells (**A**-**C**), and the results when training with MERFISH cells were calculated using a held-out set of n=28,032 cells (**D**). All error bars represent 95% confidence intervals determined by training with five bootstrapped datasets.

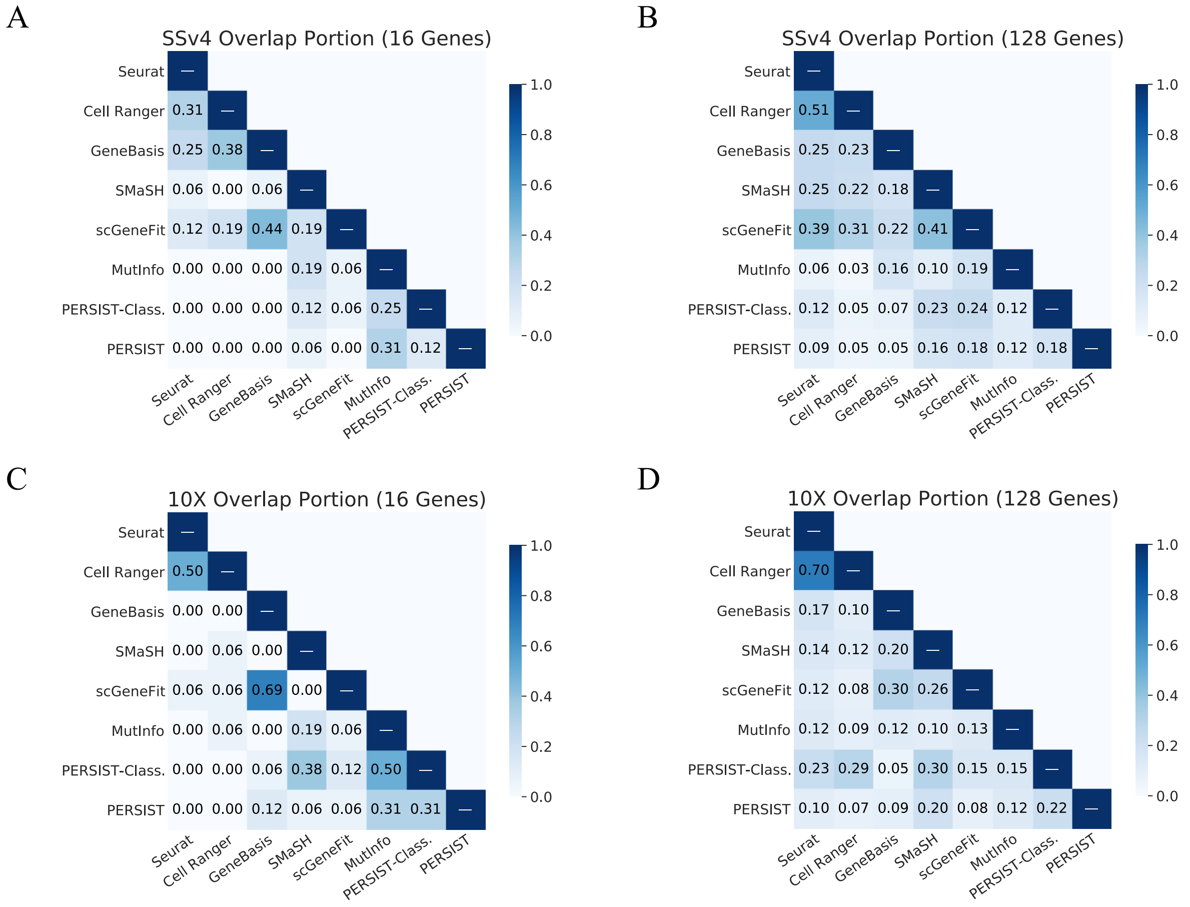

Supplementary Figure 11: **Diversity in gene panels of different sizes.** Using the SSv4 and 10X dataset, we examine the overlap between panels containing 16 and 128 genes. The genes identified by PERSIST for these panel sizes remain distinct from those selected by existing methods. **A**, Portion of overlapping genes between panels of 16 genes for the SSv4 dataset. **B**, Portion of overlapping genes between panels of 128 genes for the SSv4 dataset. **C**, Portion of overlapping genes between panels of 16 genes for the 10X dataset. **D**, Portion of overlapping genes between panels of 128 genes for the 10X dataset.

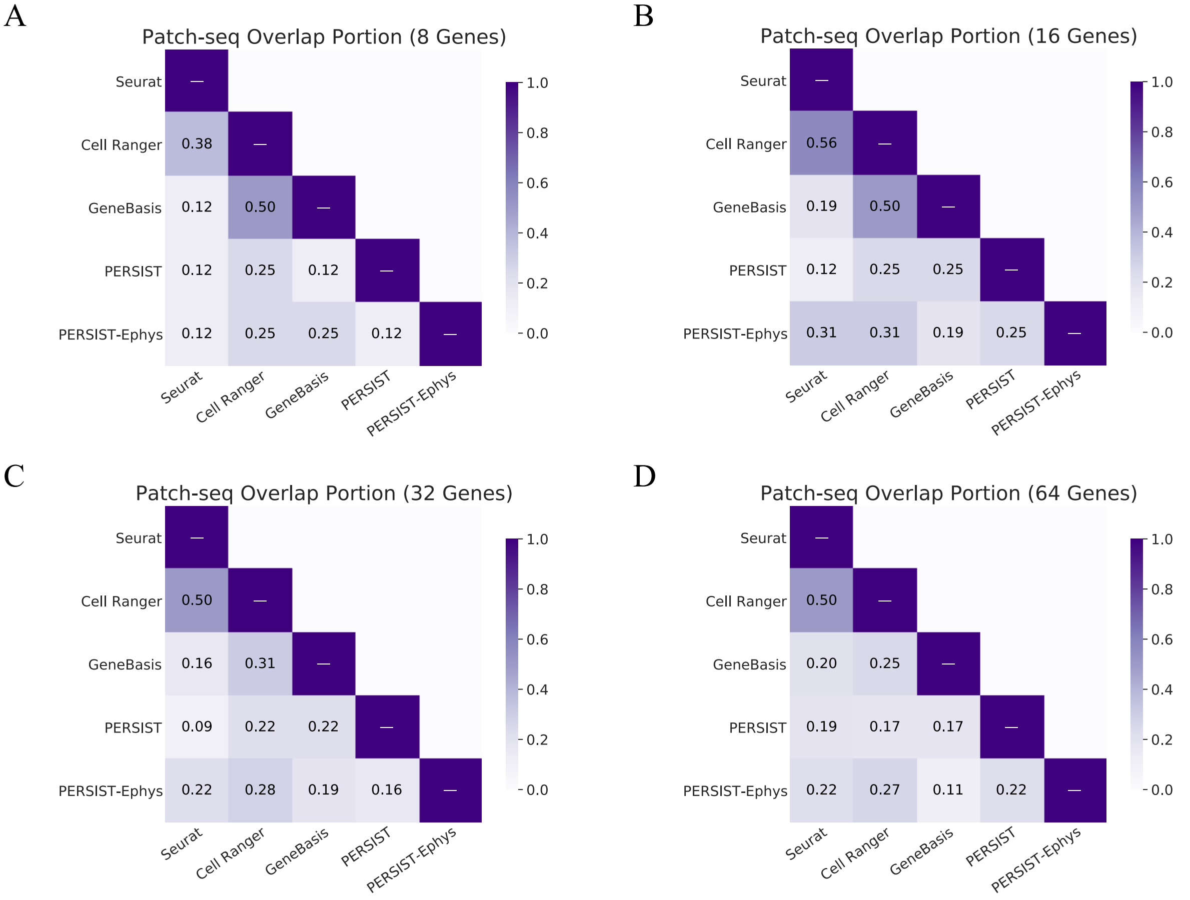

Supplementary Figure 12: **Diversity in Patch-seq gene panels.** We find that for panels of all sizes, PERSIST-Ephys selects distinct genes from the remaining methods. **A**, Portion of overlapping genes for panels of 8 genes. **B**, Portion of overlapping genes for panels of 16 genes. **C**, Portion of overlapping genes for panels of 32 genes. **D**, Portion of overlapping genes for panels of 64 genes.

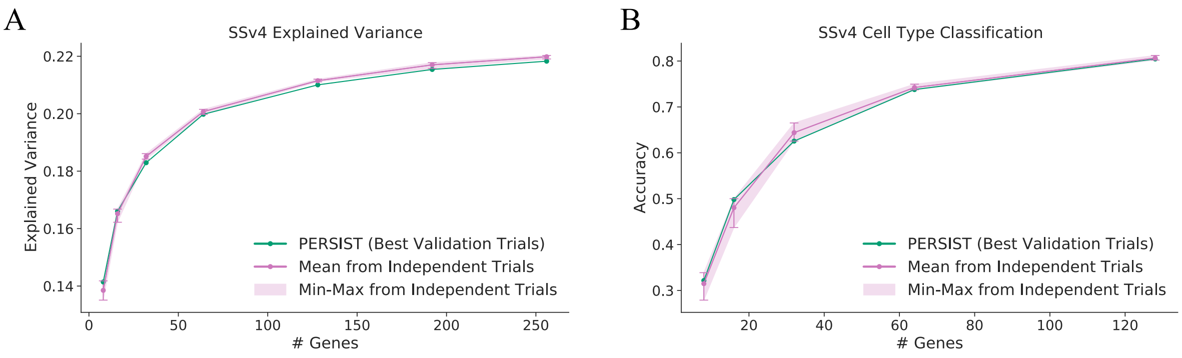

Supplementary Figure 13: **PERSIST performance variability across runs.** PERSIST can select different panels across multiple runs due to stochasticity in its training, but the variability in performance is relatively small. We compare the mean performance across five trials to the minimum and maximum performance, as well as the performance from the single trial chosen according to its validation loss. **A**, Explained variance across five trials for the SSv4 dataset. **B**, Cell type classification accuracy across five trials for the SSv4 dataset.

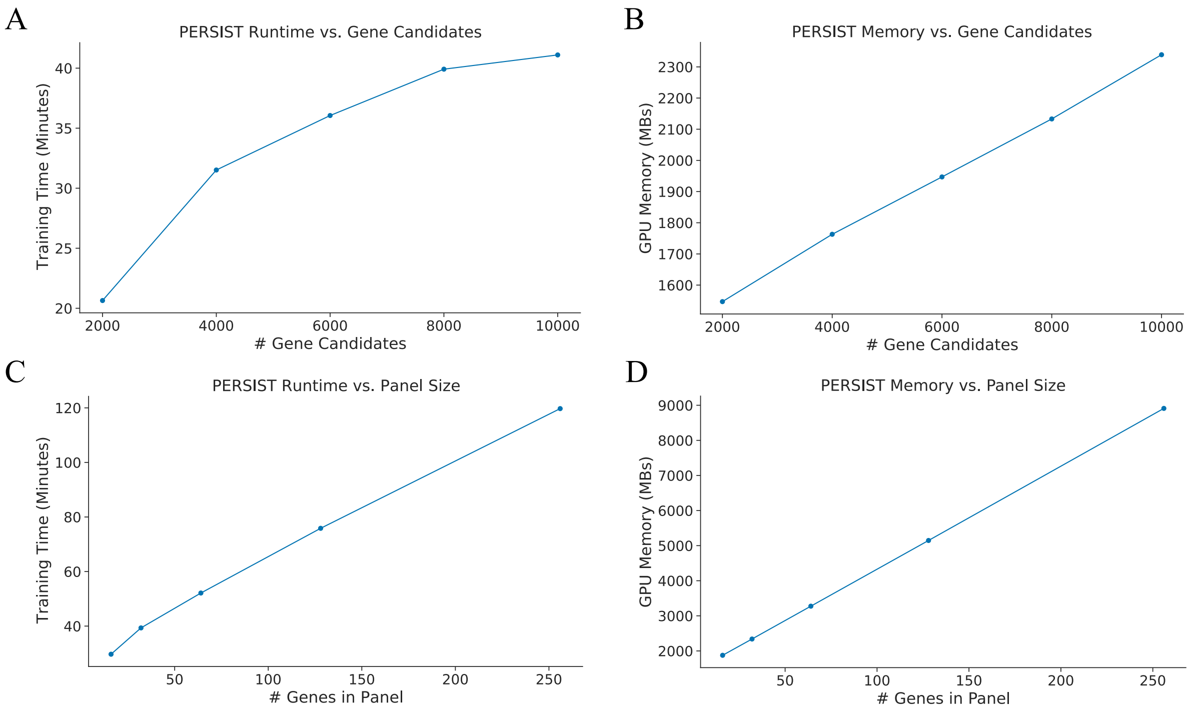

Supplementary Figure 14: **PERSIST run-time and memory usage.** We varied two parameters that affect PERSIST's computational cost, the number of candidate genes (**A**-**B**) and the number of genes in the panel (**C**-**D**), and we then measured the run-time and GPU memory usage. **A**, Run-time versus the number of candidate genes, with the number of targets fixed to 32. **B**, Memory usage versus the number of candidate genes, with the number of targets fixed to 32. **C**, Run-time versus the panel size, with the number of candidates fixed to 10,000. **D**, Memory usage versus the panel size, with the number of candidates fixed to 10,000.

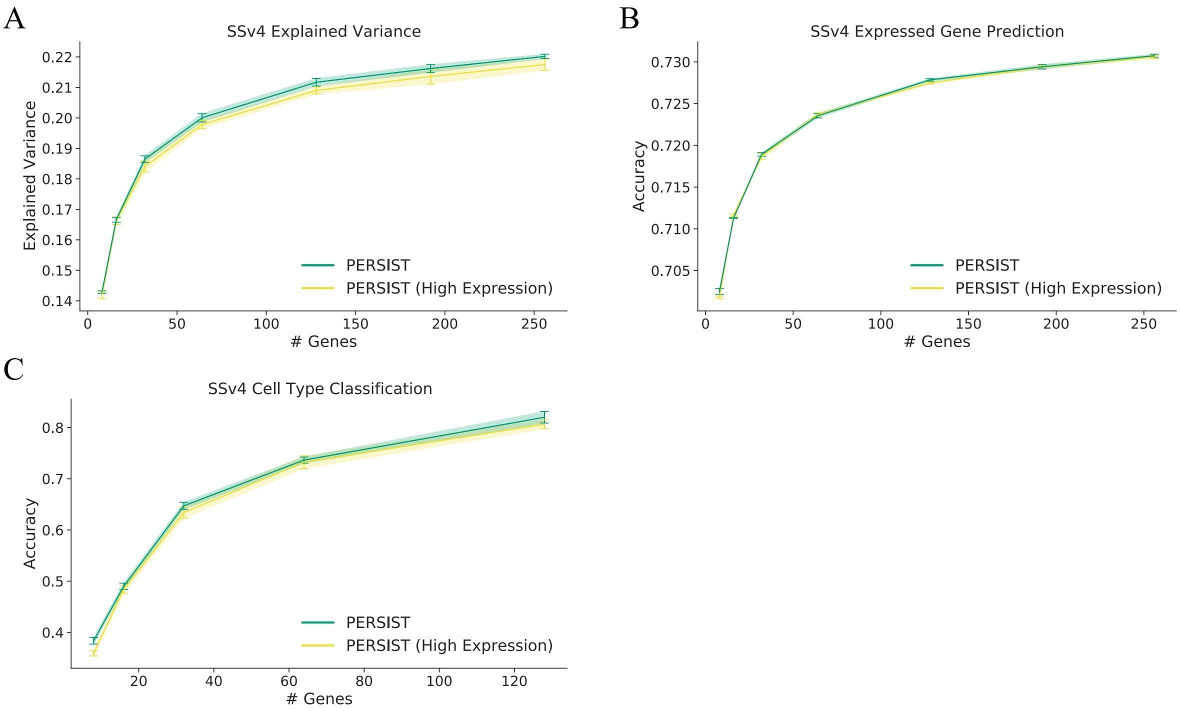

Supplementary Figure 15: **Using PERSIST with highly expressed genes only.** PERSIST can incorporate expert knowledge, for example by considering only genes with high expression (whose maximum expression level is above the median); this helps ensures that transcripts are visible during the FISH experiment. The results show that even with this restriction on the gene candidates, PERSIST can identify informative gene panels and its performance is nearly identical. **A**, Explained variance for gene panels of different sizes with the SSv4 dataset. **B**, Expressed gene prediction accuracy with the SSv4 dataset. **C**, Cell type classification accuracy with the SSv4 dataset.

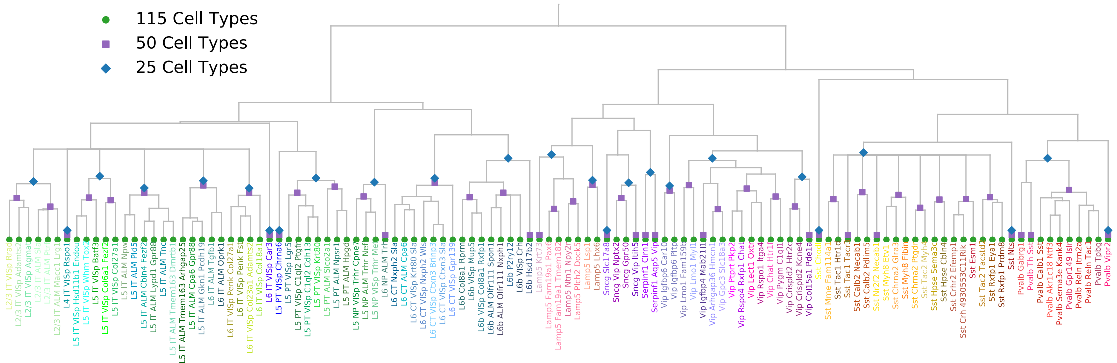

Supplementary Figure 16: **Transcriptomic hierarchy for the SSv4 V1/ALM neuronal cells.** The cell types for this dataset are defined by a transcriptomic hierarchy containing 115 total cell types. To simplify the cell type classification task, we consider merging cell types according to the transcriptomic hierarchy, yielding 50 or 25 subclasses that are more clearly defined.

Supplementary Table 1: PERSIST hyperparameter choices.

Model	Description
PERSIST (SSv4, 10X, Patch-seq)	MLP (2×128 units, ReLU activations), mbsize 128, 500 epochs
PERSIST (SSv4 Sst)	MLP (2×128 units, ReLU activations), mbsize 1024, 4,000 epochs
PERSIST (SSv4 $\rightarrow$ MERFISH)	MLP (2×128 units, ReLU activations), mbsize 128, 250 epochs
PERSIST-Classification (SSv4, 10X)	MLP (2×128 units, ReLU activations), mbsize 1024, 500 epochs
PERSIST-Ephys (PatchSeq)	MLP (2×128 units, ReLU activations), mbsize 128, 250 epochs

Supplementary Table 2: Downstream task hyperparameter choices.

Task (Dataset)	Description
Cell type classification (SSv4, 10X)	LightGBM, learning rate 0.05, 10,000 boosters (with early stopping)
Explained variance (SSv4, SSv4 Sst, 10X)	MLP (2×128 units, ReLU activations), mbsize 512, 100 epochs (with early stopping)
Expressed gene prediction (SSv4, SSv4 Sst, 10X, SSv4 $\rightarrow$ MERFISH)	MLP (2×128 units, ReLU activations), mbsize 256, 100 epochs (with early stopping)
Electrophysiological properties (Patch-seq)	MLP (2×128 units, ReLU activations), mbsize 256, 100 epochs (with early stopping)

Supplementary Table 3: Summary of datasets.

Species	Brain region	Technology	Num. Genes	Num Cells.	Annotations Used
Mouse	Primary visual (V1), anterior lateral motor (ALM)	SmartSeq v4	45,768 (10,000 in experiments)	22,160 neuronal, 2,701 Sst	Transcriptomic cell types (115 total)
Human	Motor cortex (M1)	10X	50,281 (10,000 in experiments)	72,629	Transcriptomic cell types (117 total)
Mouse	Primary visual (V1)	Patch-seq	1,252	3,411	Electrophysiological profiles (68 features)
Mouse	Primary motor (MOp)	MERFISH	258 (253 in experiments)	280,327	None
