# Supplementary material for: Predictive and robust gene selection for spatial transcriptomics
Source: Nat Commun. 2023 Apr 12;14:2091. doi: 10.1038/s41467-023-37392-1 (PMC10097645; doi:10.1038/s41467-023-37392-1)
Supplement: Supplementary file 2 — Reporting Summary [file 41467_2023_37392_MOESM2_ESM.pdf]

nature portfolio

Corresponding author(s): Uygar Sümbül, Su-In Lee

Last updated by author(s): Mar 13, 2023

Reporting Summary

Nature Portfolio wishes to improve the reproducibility of the work that we publish. This form provides structure for consistency and transparency in reporting. For further information on Nature Portfolio policies, see our Editorial Policies and the Editorial Policy Checklist.

Statistics

For all statistical analyses, confirm that the following items are present in the figure legend, table legend, main text, or Methods section.

n/a	Confirmed	
[ ]	[x]	The exact sample size (*n*) for each experimental group/condition, given as a discrete number and unit of measurement
[x]	[ ]	A statement on whether measurements were taken from distinct samples or whether the same sample was measured repeatedly
[x]	[ ]	The statistical test(s) used AND whether they are one- or two-sided<br>*Only common tests should be described solely by name; describe more complex techniques in the Methods section.*
[ ]	[x]	A description of all covariates tested
[x]	[ ]	A description of any assumptions or corrections, such as tests of normality and adjustment for multiple comparisons
[ ]	[x]	A full description of the statistical parameters including central tendency (e.g. means) or other basic estimates (e.g. regression coefficient) AND variation (e.g. standard deviation) or associated estimates of uncertainty (e.g. confidence intervals)
[x]	[ ]	For null hypothesis testing, the test statistic (e.g. *F*, *t*, *r*) with confidence intervals, effect sizes, degrees of freedom and *P* value noted<br>*Give P values as exact values whenever suitable.*
[x]	[ ]	For Bayesian analysis, information on the choice of priors and Markov chain Monte Carlo settings
[x]	[ ]	For hierarchical and complex designs, identification of the appropriate level for tests and full reporting of outcomes
[x]	[ ]	Estimates of effect sizes (e.g. Cohen's *d*, Pearson's *r*), indicating how they were calculated

Our web collection on statistics for biologists contains articles on many of the points above.

Software and code

Policy information about availability of computer code

Data collection	Our manuscript uses only existing datasets that are freely available to download online. No software was used to obtain these datasets.
Data analysis	The main algorithm in our manuscript is implemented using the PyTorch deep learning library (version 1.13.1). A full list of dependencies is available in our GitHub repository (https://github.com/iancovert/persist/blob/main/setup.py) and includes the following packages: matplotlib (3.6.2), pandas (1.5.2), scanpy (1.9.1), anndata (0.8.0), numpy (1.23.5), scikit-learn (1.2.0), h5py (3.7.0), toml (0.10.2), and tqdm (4.64.1).

For manuscripts utilizing custom algorithms or software that are central to the research but not yet described in published literature, software must be made available to editors and reviewers. We strongly encourage code deposition in a community repository (e.g. GitHub). See the Nature Portfolio guidelines for submitting code & software for further information.

Data

Policy information about availability of data

All manuscripts must include a data availability statement. This statement should provide the following information, where applicable:

- Accession codes, unique identifiers, or web links for publicly available datasets
- A description of any restrictions on data availability
- For clinical datasets or third party data, please ensure that the statement adheres to our policy

The datasets used in this work are all available online and do not require specialized accession codes. The V1/ALM SmartSeq mouse neocortex data is available at https://portal.brain-map.org/atlases-and-data/rnaseq/mouse-v1-and-alm-smart-seq. The M1 10X data is available at https://portal.brain-map.org/atlases-and-data/rnaseq/human-m1-10x. The Patch-seq data is available at https://github.com/AllenInstitute/coupledAE-patchseq. The MOp MERFISH data is available at https://

download.brainimagelibrary.org/02/26/02265ddb0dae51de/.

Human research participants

Policy information about studies involving human research participants and Sex and Gender in Research.

Reporting on sex and gender	N/A
Population characteristics	N/A
Recruitment	N/A
Ethics oversight	N/A

Note that full information on the approval of the study protocol must also be provided in the manuscript.

Field-specific reporting

Please select the one below that is the best fit for your research. If you are not sure, read the appropriate sections before making your selection.

☒ Life sciences ☐ Behavioural & social sciences ☐ Ecological, evolutionary & environmental sciences

For a reference copy of the document with all sections, see nature.com/documents/nr-reporting-summary-flat.pdf

Life sciences study design

All studies must disclose on these points even when the disclosure is negative.

Sample size	Our study used datasets consisting of different species, brain regions and sequencing technologies. The datasets were chosen to provide results that are robust across data sources, and additionally to test gene panels based on diverse quality metrics (explained variance, expressed gene prediction, cell type classification, electrophysiological property prediction, and MERFISH gene imputation). Within each dataset, our sample size was determined by the number of cells profiled in the original studies. The number of cells in each dataset is reported in the manuscript. Our choice of datasets also permits a comparison across different numbers of samples (e.g., 72,629 cells in the 10X dataset versus 2,701 Sst cells in the SSv4 dataset).
Data exclusions	We used the entirety of each dataset. For one dataset (Tasic et al., 2018), we separately evaluated our method on the entire population of neuronal cells as well as the smaller sub-population of somatostatin-expressing cells.
Replication	We ensured statistical replicability of our results by conducting multiple trials and reporting the standard deviation of each metric. Independent trials were run by boostrapping the training dataset, typically five times for each uncertainty estimate.
Randomization	Our experiments involve splitting each dataset into training, validation and test sets. Assignment to these groups is performed at random, and the train set is largest while the validation and test sets have roughly equal size. No stratification was performed.
Blinding	Knowledge of the data splits with not available during the data curation phase, as the data was not generated by the authors. To ensure fairly reported results, the test data was unseen except for when calculating final performance metrics.

Reporting for specific materials, systems and methods

We require information from authors about some types of materials, experimental systems and methods used in many studies. Here, indicate whether each material, system or method listed is relevant to your study. If you are not sure if a list item applies to your research, read the appropriate section before selecting a response.

Materials & experimental systems

n/a	Involved in the study	
☒	☐	Antibodies
☒	☐	Eukaryotic cell lines
☒	☐	Palaeontology and archaeology
☒	☐	Animals and other organisms
☒	☐	Clinical data
☒	☐	Dual use research of concern

Methods

n/a	Involved in the study	
☒	☐	ChIP-seq
☒	☐	Flow cytometry
☒	☐	MRI-based neuroimaging
