# Supplementary material for: Predictive and robust gene selection for spatial transcriptomics
Source: Nat Commun. 2023 Apr 12;14:2091. doi: 10.1038/s41467-023-37392-1 (PMC10097645; doi:10.1038/s41467-023-37392-1)
Supplement: Supplementary file 4 — Source Data [file 41467_2023_37392_MOESM4_ESM.zip › Source Data/README.docx]

Below, we provide details about the various source data files and how they were used to generate the display items in the manuscript.

The source data is organized into four directories, each of which corresponds to one of our datasets and a specific set of experiments:

- ssv4-source corresponds to the SSv4 V1/ALM dataset, which we used to evaluate expression profile reconstruction and cell type classification, and to perform ablations.
- 10x-source corresponds to the 10X dataset, which we used to evaluate expression profile reconstruction and cell type classification.
- patchseq-source corresponds to the Patch-Seq dataset, which we used to evaluate prediction of electrophysiological properties.
- merfish-source corresponds to the MERFISH dataset, which we used to evaluate the imputation of genes when training only on scRNA-seq data.

Our description of the files is organized based on these four directories, and we indicate which figures are relevant to each source file.

ssv4-source

- **ssv4_{population}_genes_{num}.csv** indicates the genes chosen by each method when training on the specified SSv4 cell subpopulation (population), and when selecting panels of the specified size (num). These results are used when generating Figure 6A, and Supplementary Figure 11A-B.
- **ssv4_{population}_reconstruction.csv** indicates the mean squared error for the expression profile reconstruction using panels selected by each method, when training on the specified SSv4 cell subpopulation (population). When these results are shown in the paper, they are post-processed to represent the explained variance: we subtract the error from the total variance, and then divide the difference by the total variance. These results are used when generating Figure 2A and 2E, Supplementary Figure 2A, Supplementary Figure 3A, and Supplementary Figure 4A.
- **ssv4_{population}_expression_{thresh}.csv** indicates the expressed gene prediction accuracy for panels selected by each method when training on the specified SSv4 cell subpopulation (population), where the genes being predicted are filtered to be those whose expression portion is not too close to 0 or 1 based on a threshold (thresh). These results were used when generating Figure 2B and 2F, Supplementary Figure 2B, Supplementary Figure 3B, Supplementary Figure 4B, and Supplementary Figure 6A-F.
- **ssv4_{population}_classification_{granularity}.csv** indicates the cell type classification accuracy using panels selected by each method when training on the specified SSv4 cell subpopulation (population), when the cell types are represented via a specified number of types (granularity). These results are used when generating Figure 3A-B and 3E-F, Supplementary Figure 2C, and Supplementary Figure 4C.
- **ssv4_computation_candidates.csv** indicates the memory and running time required to run PERSIST with different numbers of candidate genes. These results were used when generating Supplementary Figure 14A-B.
- **ssv4_computation_panelsize.csv** indicates the memory and running time required to run PERSIST with different panel sizes. These results were used when generating Supplementary Figure 14C-D.
- **ssv4_neuronal_reconstruction_variability.csv** indicates the mean squared error for the expression profile reconstruction achieved by PERSIST in five independent trials across different panel sizes, each of which involved using five bootstrapped training sets. These results were used when generating Supplementary Figure 13A.
- **ssv4_neuronal_classification_variability.csv** indicates the cell type classification accuracy achieved by PERSIST in five independent trials across different panel sizes, each of which involved using five bootstrapped training sets. These results were used when generating Supplementary Figure 13B.

10x-source

- **10x_genes_{num}.csv** indicates the genes chosen by each method for each panel size (num). These results were used when generating Figure 6B and Supplementary Figure 11C-D.
- **10x_reconstruction.csv** indicates the mean squared error for the expression profile reconstruction using panels selected by each method. When these results are shown in the paper, they are post-processed to represent the explained variance: we subtract the error from the total variance, and then divide the difference by the total variance. These results are used when generating Figure 2C, Supplementary Figure 3C, and Supplementary Figure 4D.
- **10x_expression_{thresh}.csv** indicates the expressed gene prediction accuracy for panels selected by each method, where the genes being predicted are filtered to be those whose expression portion is not too close to 0 or 1 based on a threshold (thresh). These results were used when generating Figure 2D, Supplementary Figure 3D, Supplementary Figure 4E, and Supplementary Figure 6G-I.
- **10x_classification.csv** indicates the cell type classification accuracy using panels selected by each method. These results are used when generating Figure 3C-D and Supplementary Figure 4F.

patchseq-source

- **patchseq_genes_{num}.csv** indicates the genes chosen by each method for each panel size (num). These results were used when generating Supplementary Figure 13. The expression levels of these genes for PERSIST-Ephys were also used when generating Figure 4B.
- **patchseq_reconstruction.csv** indicates the mean squared error when attempting to reconstruct the electrophysiological features provided with the dataset. These results were used when generating Figure 4A.

merfish-source

- **merfish_{population}_genes_{num}.csv** indicates the genes chosen by each method for each number of genes (num) when using SSv4 cells from the specified subpopulation (population). These results were not directly used when creating any display figures.
- **merfish_{population}_expression_thresh.csv** indicates the gene imputation accuracy for each method when panels were chosen using the specified SSv4 subpopulation (population), and when MERFISH genes were binarized using a threshold matching the quantile of the corresponding SSv4 cells. These results were used when generating Figure 5B-C and Supplementary Figure 10 A-D.
- **merfish_{population}_expression_zero.csv** indicates the gene imputation accuracy for each method when panels were chosen using the specified SSv4 subpopulation (population), and when MERFISH genes were binarized using a zero threshold. These results were used when generating Supplementary Figure 10C.
- **merfish_{population}_expression_merfish_thresh.csv** indicates the gene imputation accuracy for each method when panels were chosen using the specified SSv4 subpopulation (population), when MERFISH genes were binarized using a threshold matching the quantile of the corresponding SSv4 cells, and when using MERFISH as the training data. These results were used when generating Supplementary Figure 10D.
